# Skull base reconstruction: A question of flow? A critical analysis of 521 endoscopic endonasal surgeries

**DOI:** 10.1371/journal.pone.0245119

**Published:** 2021-03-15

**Authors:** Giuseppe Di Perna, Federica Penner, Fabio Cofano, Raffaele De Marco, Bianca Maria Baldassarre, Irene Portonero, Diego Garbossa, Luca Ceroni, Giancarlo Pecorari, Francesco Zenga

**Affiliations:** 1 Department of Neuroscience "Rita Levi Montalcini", Neurosurgery Unit, University of Turin, Turin, Italy; 2 Spine Surgery Unit, Humanitas Gradenigo Hospital, Turin, Italy; 3 Psychological Sciences and Techniques, Psychology Department, University of Turin, Turin, Italy; 4 Department of Surgical Sciences, ENT Surgery Unit, University of Turin, Turin, Italy; AUSL della Romagna, ITALY

## Abstract

**Introduction:**

Post-operative CSF leak still represents the main drawback of Endoscopic Endonasal Approach (EEA), and different reconstructive strategies have been proposed in order to decrease its rate.

**Objective:**

To critically analyze the effectiveness of different adopted reconstruction strategies in patients that underwent EEA.

**Materials and methods:**

Adult patients with skull base tumor surgically treated with EEA were retrospectively analyzed. Data recorded for each case concerned patient demographics, type of surgical approach, histotype, anatomical site of surgical approach, intra-operative CSF leak grade (no leak (INL), low flow (ILFL), high flow (IHFL)), reconstructive adopted strategy, Lumbar Drain positioning, post-operative CSF leak rate and intra/post-operative complications.

**Results:**

A total number of 521 patients (January 2012-December 2019) was included. Intra-operative CSF leak grade showed to be associated with post-operative CSF leak rate. In particular, the risk to observe a post-operative CSF leak was higher when IHFL was encountered (25,5%; Exp(B) 16.25). In particular, vascularized multilayered reconstruction and fat use showed to be effective in lowering post-operative CSF leaks in IHFL (p 0.02). No differences were found considering INL and ILFL groups. Yearly post-operative CSF leak rate analysis showed a significative decreasing trend.

**Conclusion:**

Intra-operative CSF leak grade strongly affected post-operative CSF leak rate. Multilayer reconstruction with fat and naso-septal flap could reduce the rate of CSF leak in high risk patients. Reconstructive strategies should be tailored according also to the type and the anatomical site of the approach.

## Introduction

Midline skull base tumors are rare lesions, characterized by different grades of malignancy and recurrence rate [1]. The oncological goal of achieving a gross total removal (GTR), while preserving neuro-endocrinological functions, has represented a major challenge for skull base surgeons during the past years [2–4]. Nowadays, Endoscopic Endonasal Approach (EEA) is proving to be a real game changer as well as the best way to treat midline skull tumors, such as craniopharyngiomas, pituitary adenomas and chordomas [2, 3, 5–7].

Postoperative cerebro-spinal fluid (CSF) leaks still represent the main drawback of the endoscopic endonasal approach [7–9]. Its rate is strictly related to the possibility of a proper reconstruction of the skull base defect, in order to re-create the physiological separation between intracranial and extracranial spaces and to avoid complications like meningitis and pneumocephalus [10]. In the last years, many steps forward have been made in the amelioration of reconstruction techniques, lowering the post-operative CSF leaks (POL) rates (2,6%–8,9%) reported in previous papers (10%–40%) [11, 12].

The aim of this study is to critically analyze the effectiveness of various adopted reconstruction strategies on the post-operative CSF rate in patients undergoing EEA in a single center experience (2012–2019), operated by a consolidated ENT-Neurosurgical team.

The secondary goals of this study are to analyze the relationships between the post-operative CSF leak and (1) the type of surgical approach, (2) the anatomical site of the approach, (3) the grade of intra-operative CSF leak (IOL), (4) the history of previous trans-sphenoidal surgery and radiotherapy, (5) the peri-operative lumbar drain (LD) positioning and (6) the surgeon’s experience and learning curve.

## Patients and methods

This is a retrospective observational study analyzing data of 566 patients affected by skull base neoplasms who underwent EEA between January 2012 and December 2019.

Inclusion criteria were: 1) history of skull base surgery, in adults (>17 years), 2) performed with 3D-EEA and 3) at least a 3-month follow-up. Exclusion criteria were: 1) Not complete availability of patient’s clinical and radiological data; 2) the presence of complex CVJ malformations; 3) the absence of the Senior Surgeon (FZ) during the procedure.

Data were extracted from a prospective collected on-line and shared database, filled during patient’s hospitalization and follow-up, and included: age, sex, tumor histology, type of endoscopic endonasal approach (standard or extended), location of the osteo-dural defect according to the site of the approach, presence of intra-operative CSF leakage, different reconstruction techniques, perioperative lumbar drain (LD) positioning, postoperative CSF leakage rate, occurrence and type of intra and post-operative complications and occurrence of radiotherapy before surgery. Clinical and radiological data were obtained at the time of admission and at the follow-up evaluation by fully trained neurosurgeons.

In particular, data have been collected in an on-line database (https://molinette.codize.app) that is shared among physicians belonging to the authors’ institution; the database itself is not publicly available according to the privacy policy of the authors’ institution. Moreover, data have been anonymized before being accessed and analyzed.

### Type and anatomical site of the approach

All of the patients were treated using a 3DHD endoscope. Surgery was performed using a “two-nostril—four-hand” technique, previously described [13, 14].

Patients were divided into two different groups according to the type and the anatomical site of the approach: standard or expanded approach. Standard approach consisted of entirely trans-sellar corridor after anterior sphenoid sinus wall removal and sellar floor opening.

Expanded approach consisted of sagittal or coronal extension of the standard one. Unilateral or bilateral posterior ethmoidectomy, different types of maxillectomy and eventually pterygoid removal provided extension in a coronal plane. The extension on the sagittal plane, instead, included a trans-tuberculum, trans-planum sphenoidale and/or trans-ethmoidal approach. The extended trans-clival approach allowed access to the posterior fossa.

### CSF leak grading

CSF leaks occurring during surgical procedures were divided into 3 groups: intra-operative no CSF leaks (INL), intra-operative low flow CSF leaks (ILFL) and intra-operative high flow CSF leaks (IHFL). As commonly accepted [15, 16], ILFL included dural opening with minimal flow observed or no involvement of basal cisterns or ventricle, while basal cisterns or ventricular opening defined the IHFL group. Moreover, among patients belonging to the INL group, the presence of thin arachnoid membranes or small dural tears, the early descent of supra-sellar cisterns and the coexistence of independent risk factor for POL and meningitis (e.g. high BMI, older age) [10] identified the INL* subgroup (Table 1).

**Table 1:** Materials and methods. Intra-operative CSF leak grading system, reconstruction methods and reconstructive strategies.

**Intra-operative CSF Leak Grading**	
**No CSF leak (INL)**	No intracranial opening or small osteo-dural defect without intraoperative leakage
**Low flow CSF leak (ILFL)**	Small osteo-dural opening and minimal leakage (no basal cisterns or ventricular opening)
**High flow CSF leak (IHFL)**	Large osteo-dural defect with basal cisterns or ventricular opening
**Reconstruction methods**	
**Synthetic Dural Substitute**	1, 2 or 3 layers. (Redura, Duragen and Lyoplant Onlay differently adopted)
**Fat**	Abdominal autologous
**Ileo tibial tract (ITT)**	Harvested from lateral side of the thigh
**Mucosal flap**	Harvested from posterior wall of sphenoid sinus
**Free graft of nasal mucosa**	Harvested from inferior or middle turbinate
**Naso-Septal Flap (NSF)**	Harvested from the septal mucoperiosteum and mucoperichondrium preserving the patency of sphenopalatine artery
**Inverted U-shaped rhino-pharyngeal mucosal flap**	Harvested from the rhino-pharyngeal mucosa
**Reconstructive Strategies**	
INL*	**Type 1: Minimal Reconstruction** Sponge and fibrin glue to cover the defect Sphenoid sinus mucosal flap when preserved
**ILFL**	**Type 2: Standard Reconstruction** Synthetic dural substitute or ileo-tibial tract (ITT) Autologous abdominal Fat *Mucosal flap or free graft could be added*
**IHFL**	**Type 3: Sandwich multilayered reconstruction** Synthetic Dural Substitute or ITT *positioned as inlay (inside the dura)* Autologous abdominal fat *filling the empty dead space* Synthetic Dural Substitute or ITT *positioned as onlay (between dura and inner bone)* Autologous Abdominal Fat *covering irregular edges of the reconstruction* NSF or Inverted U shape rhino-pharingeal flap
*INL with augmented POL risk (high BMI, elderly, early prolapse of supra-sellar cisterns, thin arachnoid membrane)	**Incomplete type 2 or type 3 reconstruction**

INL: No CSF leak; ILFL: Intra-operative Low Flow CSF Leak; IHFL: Intra-operative High Flow CSF Leak.

### Techniques and strategies of skull base reconstruction

Skull base reconstruction strategy was divided according to 1) size of defect; 2) anatomical site of surgical approach; 3) grade of IOL and 4) type of tumor. Available methods to plan proper reconstruction strategies were (Table 1): A) Synthetic dural substitute (Redura, Lyoplant Onlay, Duragen) used in a inlay or onlay fashion, albeit different matrixes were not individually analyzed; B) Autologous abdominal fat; C) Ileo tibial tract (ITT), harvested from the lateral side of thigh; D) sinus mucosal flap, harvested from the posterior wall of sphenoid sinus and positioned on the defect; E) Free graft of nasal mucosa from inferior or middle turbinate; F) Naso-septal flap (NSF) harvested from the septal muco-periosteum and muco-perichondrium, preserving the patency of sphenopalatine artery [17]; G) Inverted U-shaped rhino-pharyngeal mucosal flap harvested detaching mucosa and muscles from the lower clivus and cranio-vertebral junction (CVJ). No materials like bone, cartilage or rigid artificial substitute were used.

**Fig 1:**
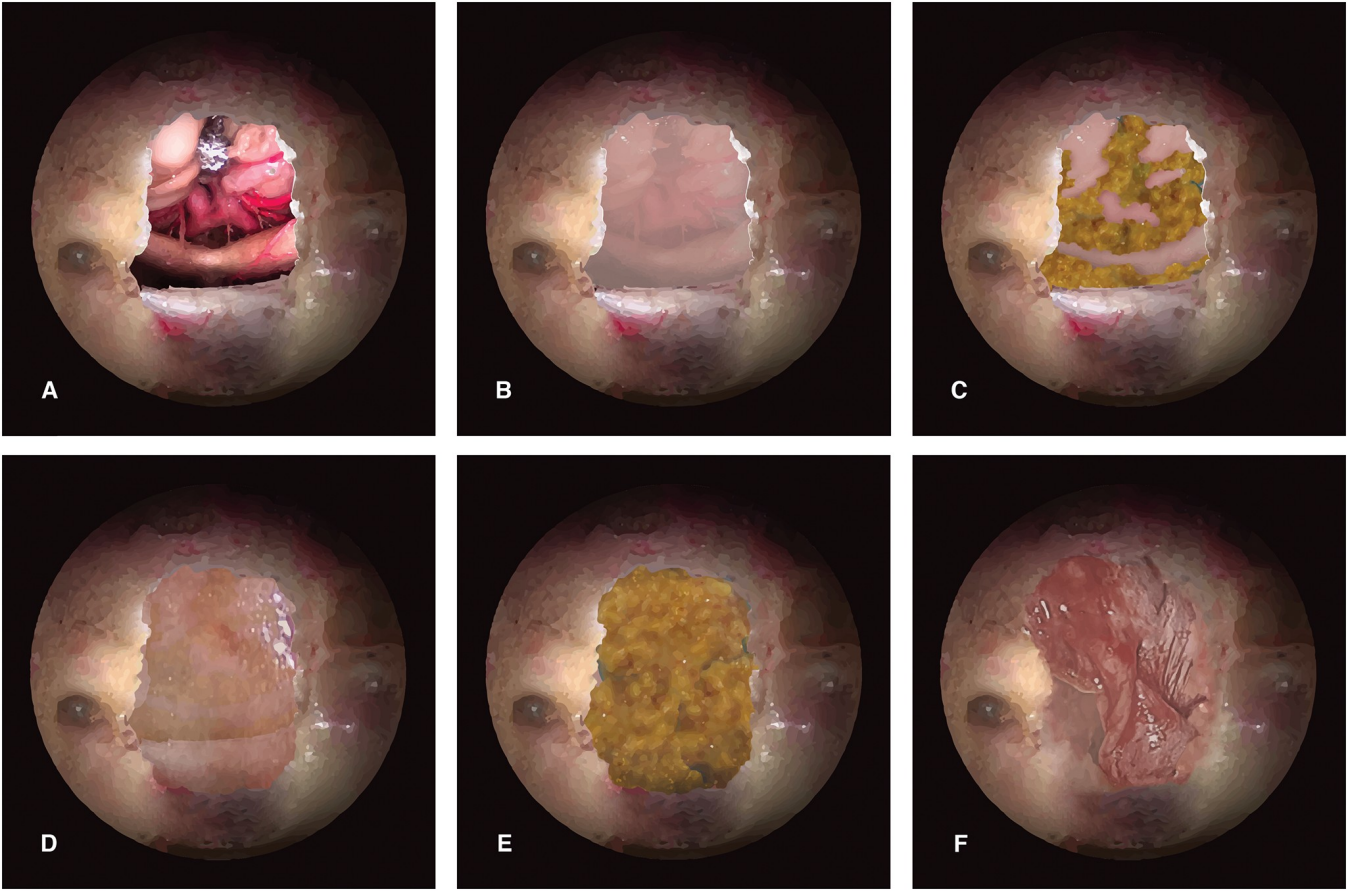
The “sandwich” multilayer closure. Illustrations (A-F) depict a step-by-step multilayer closure after transtuberculum-transplanum approach for skull base tumors, such as meningioma or craniopharyngioma. A, shows a detail of the neurovascular structures that can be seen in this type of approach, when the tumor is removed; B, a first layer of derived dural is positioned “inlay”, with its edges under the dura; C, an autologous fat graft is collected at the abdomen and it is used to fill the dead spaces (it is considered intraosseous); D, a second layer of collagen derived matrix is positioned “onlay”, between the dura and bone edges, or over the bone defects; E, another layer of abdominal fat graft is positioned (extraosseous); F, finally NSF, harvested at the beginning of the surgery, is carefully rotated on the skull base defect, where the mucosa has already been removed to avoid mucocele formation.

The strategies adopted for reconstruction in this series were summarized in 3 major groups (Table 1):

1. Minimal Reconstruction: in cases with a small intracranial opening without CSF leakage, a sponge was used to fill the surgical cavity fixed with fibrin glue. Mucosal flap from sphenoid sinus could have also been used in this strategy (when/if preserved).
2. Standard Reconstruction: dural synthetic substitutes were used for small intracranial openings and for ILFL. In this group autologous abdominal fat and inferior or middle turbinate mucosa was often used respectively to fill the cavity and to cover the defect. Fibrin glue was then used to adhere the dural substitute and fat together. Additionally, patients belonging to the INL* subgroup, who underwent fat and/or reconstruction, have been included in this group as “incomplete type 2 reconstruction”.
3. “Sandwich” Multilayered reconstruction (Fig 1): this strategy was used for large defect, highly risky sites in the surgical approach (anterior fossa and clivus) and IHFL (Fig 1A). Also, patients belonging to the INL* subgroup, in which a pedicled flap reconstruction was performed, have been included in this group as “incomplete type 3 reconstruction”. The first layer consisted in an inlay-positioned synthetic dural substitute or ITT (Fig 1B); then, autologous abdominal fat was placed on the previous layer to fill the empty space (Fig 1C); finally, a second membranous layer was positioned in an onlay fashion, or between the dura and inner bone surface (Fig 1D). At this point, autologous fat was used to cover the lateral edges of the reconstruction outside the bone, tailoring the closure especially in cases with irregular osteo-dural defects (Fig 1E). Every layer was secured with fibrin glue. NSF or rhino-pharyngeal flap, according to the location of the approach, was then rotated on the skull base defect and tightened with fibrin glue (Fig 1F). A paramount step was to fill the empty dead space. The NSF needed to be laid with the muco-perichondral side in direct contact with posterior wall of the sphenoid sinus, in order to avoid a subsequent mucocele formation. Attention was paid to flatten any bony asperity. Poly-vinyl sponges were used to take in site the flap and to pack nasal cavities. Foley catheter was used to take in place the rhino-pharyngeal flap.

Nonetheless, although the aforementioned strategies were adopted in the majority of cases, several not standardizable combinations of available materials could have been used for borderline cases (incomplete type 2 and 3 reconstructions).

### Lumbar drain positioning criteria

The type of surgical approach and consequent probability for high flow CSF leak represented the criteria for Lumbar Drain (LD) positioning. More specifically, it was used in every expanded approach with osteo-dural defect in the anterior cranial fossa, in the clival region and in the sellar region, and/or when supra-sellar extension was needed (trans-tuberculum and trans-planum approaches). LD was positioned before surgery, under general anesthesia and opened only after surgery.

### Statistical analysis

Descriptive statistics were reported as mean and standard deviation for continuous variables. Comparison of proportions were performed with Chi-squared test for categorical variables and, when needed (>20% of values < = 5 and/or presence of values<1), with Cramer’s Phi and V coefficients to verify association between variables. Relationships between dependent and independent variables were evaluated using logistic regression. Moreover, 3 casual extractions from the database were made in order to obtain parametric variables. Statistical significance was defined with a p-value < 0.05. All statistical analyses were performed using SPSS Statistics software.

## Results

A total of 521 patients (M: F 283:238, 54.3%:45.7%) were collected after retrospective evaluation of inclusion/exclusion criteria. Descriptive data of the study population and surgical results are reported in Table 2. The mean age of patients was 54.3 years (SD 15.96; range from 17 to 86). Macroadenoma (63.5%), chordoma (5%) and craniopharyngioma (4.6%) were the most frequent tumors. An expanded approach was performed in 109 cases (20.9%), while the standard approach was carried out in 412 patients (79.1%). The global rate of POL was 5.2%, (27 patients). Concerning the different reconstruction techniques adopted, type 1 was adopted in 289 patients (55.5%), type 2 in 124 cases (23.8%), and type 3 in 108 patients (20.7%).

**Table 2:** Descriptive results.

	n	%
**Sex**	M:F 283:238	54.3%: 45.7%
**Age** (mean value)	54.3 (range 10–86)	
**ASA** (mean value)	1.8 (range 1–4)	
**Type of Tumor**		
Microadenoma	59	11.3%
Macroadenoma	331	63.5%
Craniopharyngioma	24	4.6%
Chordoma	26	5%
Meningioma	11	2.1%
Chondrosarcoma	7	1.3%
Other tumors	63	12.1%
**Surgical Approach**		
Standard	412	79.1%
Expanded	109	20.9%
**Anatomical site of surgical approach**		
Anterior (Trans-planum, Trans-tuberculum, ethmoid)	62	11.9%
Posterior (Trans-clivus, CVJ, trans-petrous)	55	10.6%
Sellar/Supra-sellar	404	77.5%
**IOL grade**		
INL	391	75%
ILFL	76	14.6%
IHFL	51	9.8%
**Reconstruction Strategy**		
Type 1 (sponge, fibrin glue, (mucosal flap))	289	55.5%
Type 2 (multilayer: dural substitute and fat)	124	23.8%
Type 3 (multilayer + vascularized flap)	108	20.7%
**LD Positioning**	63	12.1%
**Second Surgery**	77	14.8%
**Previous Radiotherapy**	9	1.7%
**Post-operative CSF leak**	27	5.2%

IOL: Intra-Operative Leak; INL: No CSF leak; ILFL: Intra-operative Low Flow CSF Leak; IHFL: Intra-operative High Flow CSF Leak; LD: Lumbar Drain.

**Fig 2:**
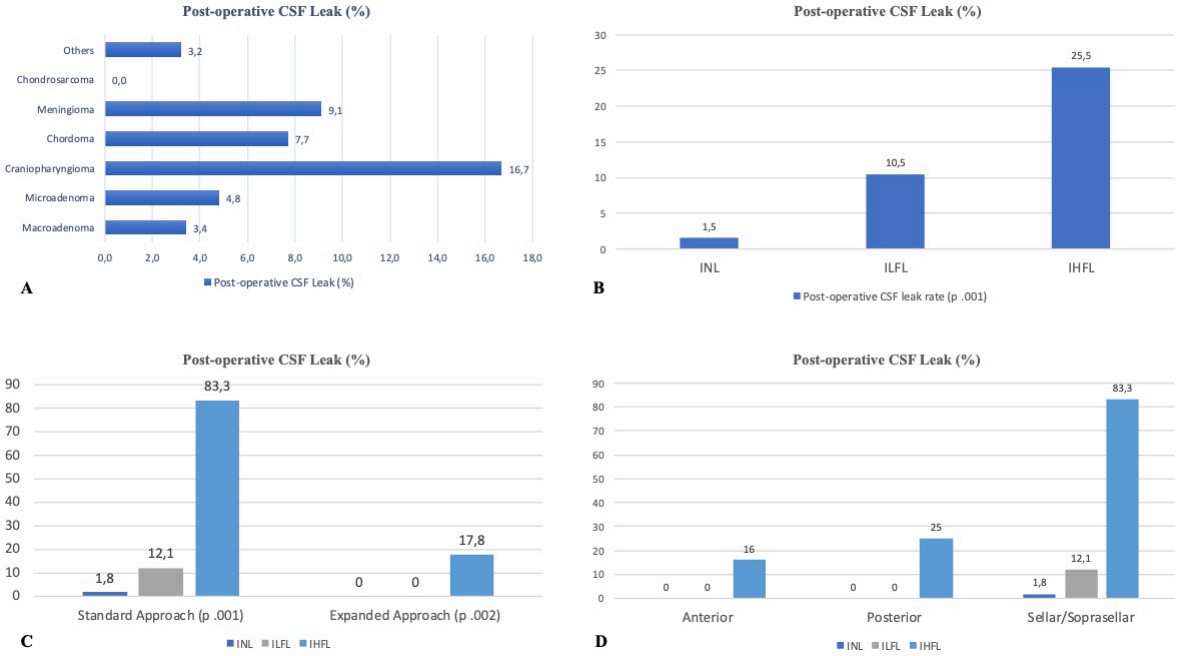
A, Post-operative CSF leak rate observed in different tumors, B, post-operative CSF leak analyzed in different type of surgical approach, according to different IOL groups; C, post-operative CSF leak analyzed in different IOL groups, D, post-operative CSF leak analyzed in different anatomical site, according to different IOL groups.

Analyzing different types of tumors, a statistically significant association between POL and craniopharyngioma diagnosis was registered (CSF leak rate 16,7%, p.045). (Fig 2).

Associations between POL rate and the grading of IOL, the type and the site of surgical approach are reported in Table 3. POL rate is reported as 1.5% in INL patients, 10.5% in ILFL group, while the IHFL group showed 25.5% of POL rate. These results reached a statistical significance (p.001). As for the type of surgical approach, POL rate was reported in 19 cases (4.6%) among patients in the standard approach group and in 8 patients (7.3%) in the expanded approach group (p.253) (Table 3). A multivariate logistic regression was performed in order to find associations between POL rate and type of surgical approach, anatomical site of the approach and IOL grade (Table 4). Logistic regression showed statistically significant associations between POL rate and the entity of IOL (Nagelkerke R-squared.062, Exp(B) 50.86, p.002). Moreover, multivariate logistic regression for different grades of IOL reported progressively higher risk for different grades (INL: Exp(B) 0.016, p.002; ILFL: Exp(B) 5.827, p.040; IHFL: Exp(B) 16.25, p.001) (Table 4).

**Table 3:** Post-operative CSF leak rate differentiated according to type of approach, site of approach and grade of intra-operative CSF leak.

**Type of approach**				
	Post-operative CSF leak rate (%)	No post-operative CSF leak rate (%)		
Standard	19 (4.6)	393 (95.4)		
Expanded	8 (7.3)	101 (92.7)		
			Chi-squared	.253
			Phi	.253
			Cramer V	.253
**Anatomical site of approach**				
	Post-operative CSF leak rate (%)	No post-operative CSF leak rate (%)		
Anterior	6 (9.7)	56 (90.3)		
Posterior	2 (3.6)	53 (96.4)		
Sellar/Sopra-sellar	19 (4.7)	385 (95.3)		
			Chi-squared	.222
			Phi	.222
			Cramer V	.222
**IOL grade**				
	Post-operative CSF leak rate (%)	No post-operative CSF leak rate (%)		
INL (INL*)	6 (1.5) (2 (1.9))	386 (98.5) (102 (98.2))		
ILFL	8 (10.5)	68 (89.5)		
IHFL	13 (25.5)	38 (74.5)	Chi-squared	**.001**
			Phi	**.001**
			Cramer V	**.001**

IOL: Intra-Operative Leak; INL: No CSF leak; ILFL: Intra-operative Low Flow CSF Leak; IHFL: Intra-operative High Flow CSF Leak.

**Table 4:** Multivariate Logistic regression considering post-operative CSF leak rate (dependent variable) and type of approach, anatomical site of surgical approach, sex, intra-operative CSF leak grade, type of tumor and second surgery (independent variables), and univariate logistic regression for single grades of intra-operative CSF leak.

**Multivariate Binomial Logistic Regression**			
**Variables**	**Nagelkerke R-squared**	**Exp(B)**	**pValue**
Type of Approach	**.602**	0.002	.125
Anatomical site of approach		0.149	.345
Sex		1.430	.685
IOL grade		50.860	**.002**
Type of Tumor		0.795	.640
Second Surgery		0.059	.045
**Univariate Binomial Logistic Regression**			
**IOL grade**	**Nagelkerke R-squared**	**Exp(B)**	**pValue**
INL	**.301**	16.25	**.002**
ILFL	**.121**	5.827	**.040**
IHFL	**.550**	0.016	**.001**

IOL: Intra-Operative Leak; INL: No CSF leak; ILFL: Intra-operative Low Flow CSF Leak; IHFL: Intra-operative High Flow CSF Leak.

Patients were stratified in different categories according to the statistical weight of the grade of IOL. In the standard approach group, POL rate was 1.8% when IFL was encountered, 12.1% (8 patients) when ILFL was reported and 83.3% (5 patients) in cases of IHFL (p.001). In the expanded approach group, POLs rate was 0% when IFL or ILFL was registered, reaching 17.8% (8 patients) when IHFL was encountered during surgery (p.002) (Fig 2).

No statistically significant differences between post-operative CSF rate and type of reconstruction were found after stratification in patients with INL or ILFL. Moreover, among patients belonging to INL leak, 102 (26.8%) underwent incomplete type 2 or type 3 reconstruction. In this subgroup POL rate was 1.9% and there was no significative difference compared to POL rate in the type 1 reconstruction (1.4%).

Conversely, while analyzing patients with IHFL and their POL rate, statistically significant differences were found comparing different reconstructive strategies. POL rate resulted 4.3% in type 3 reconstruction group, while it was 42.9% in patients where type 1 or type 2 strategies were adopted (p.002). Moreover, univariate logistic regression showed significant influence of fat used in type 3 reconstruction (Exp(B) 0.58; p.001) (Table 5).

**Table 5:** Post-operative CSF leak rate among different IHFL subgroups: Patients with type 3 reconstruction; use of different number of dural layers; use of fat and lumbar drain positioning.

**Type 3 Reconstruction in IHFL group**				
	Post-operative CSF leak rate (%)	No post-operative CSF leak rate (%)		
No type 3 reconstruction	12 (42.9)	16 (57.1)		
Type 3 reconstruction	1 (4.3)	22 (95.7)		
			Chi-squared	**.002**
			Phi	**.002**
			Cramer V	**.002**
**2 Dural Substitutes Multilayer Reconstruction in IHFL group**				
	Post-operative CSF leak rate (%)	No post-operative CSF leak rate (%)		
No 2 layers reconstruction	12 (32.4)	25 (67.6)		
2 layers reconstruction	0 (0)	13 (100)		
			Chi-squared	**.010**
			Phi	**.010**
			Cramer V	**.010**
**Univariate Logistic Regression**		Nagelkerke R-squared	Exp (B)	p value
Fat		0.318	0.058	**0.01**
**Fat positioning in IHFL Group**				
	Post-operative CSF leak rate (%)	No post-operative CSF leak rate (%)		
No fat positioning	12 (42.9)	16 (57.1)		
Fat positioning	1 (4.3)	22 (95.7)		
			Chi-squared	**.010**
			Phi	**.010**
			Cramer V	**.010**
**Lumbar Drain Positioning in IHFL group**				
	Post-operative CSF leak rate (%)	No post-operative CSF leak rate (%)		
No LD positioning	5 (29.4)	12 (70.6)		
LD positioning	8 (23.5)	26 (76.5)		
			Chi-squared	.650
			Phi	.650
			Cramer V	.650

INL: No CSF leak; ILFL: Intra-operative Low Flow CSF Leak; IHFL: Intra-operative High Flow CSF Leak.

Focusing on the role of fat grafts, results confirmed a paramount difference between the presence/absence of IHFL: a statistical significance was reported in the IHFL group (4.3%with fat vs 42.9% without fat; p.001) (Table 5).

Moreover, in the same IHFL group, a significant difference of POL rate was found in patients when 2 dural substitutes were used (0%) compared to patients with different multilayer combination (32.4%) (p.010) (Table 5).

LD positioning was analyzed according to different IOL grade. No significant statistical differences were found in the ILFL nor the IHFL groups (Table 5).

**Fig 3:**
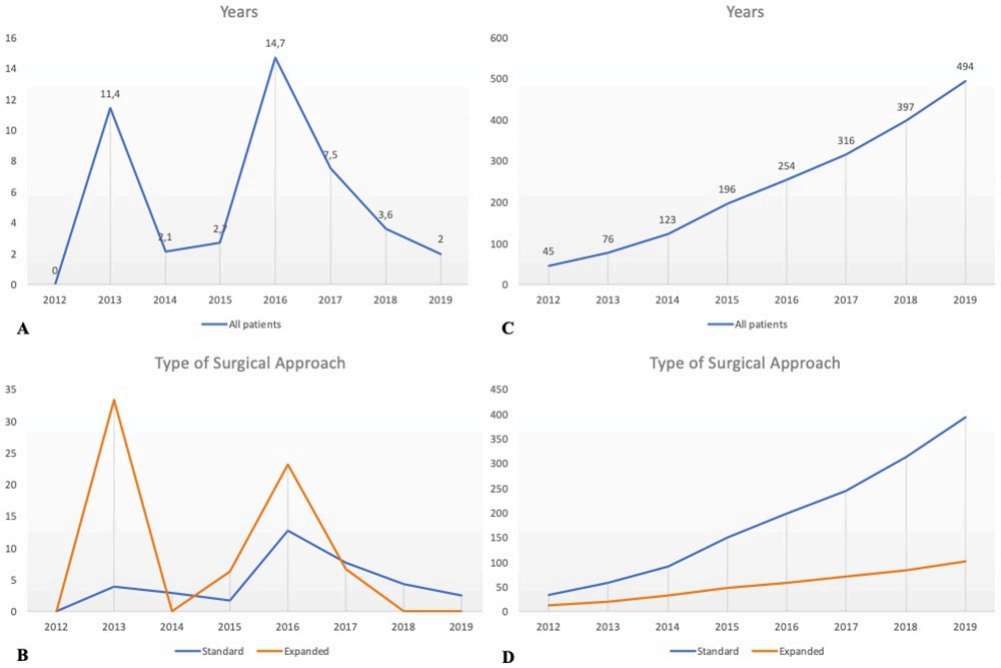
A population analysis over the years. A graphical analysis of postoperative CSF-leak is represented in Graphs A and B; graphs C and D show the trend over time for no post-operative leak.

Finally, POL rate was considered through the years showing a decreasing trend in the last 4 years, from the highest value of 14.7% reported in 2016 to the lowest value of 2% registered during 2019 (p.020) (Fig 3).

## Discussion

The endoscopic endonasal approach (EEA) have evolved during the years, allowing the treatment of complex skull base lesions [7]. Minimal invasiveness due to the absence of brain retraction, the development of surgical technique and technological innovation, allow these approaches to be widely spread [3, 18]. Additionally, recent papers have emphasized the adherence of endoscopic procedures to oncological principles in the treatment of benign and malign skull base tumors [2].

Nevertheless, POL related complications such as meningitis and pneumocephalus still remain the main drawbacks of EEA [18–20]. Therefore, skull base reconstruction should be considered a crucial part of the procedures and the real challenge in the evolution of EEA [7, 10, 18, 21].

In order to achieve a good reconstruction, the primary goal should be re-creating a physiological separation between intracranial and extracranial spaces [22]. To achieve rapid recovery and protecting the exposed neuro-vascular structures should be the secondary goals [3, 7].

### Reconstruction techniques and POL rate

The development of different materials and different strategies for reconstruction resulted in reduction of POL rate, from the higher values reported in the first papers (10%–40%) [17, 23–35], to the conspicuously lower rate described more recently (from 2,6% to 8,9%) [11, 12, 18]. In this study the overall POL rate was 5.2%, thus confirming the encouraging trend described by the evidence.

Many reconstructive methods and different materials have been reported in the literature [8, 15] but it is likely that the introduction of NSF and the principle of multilayered closure constituted the real game-changer in skull base reconstruction.

Hadad et al. described in 2006 a novel technique for reconstruction of skull base defect using a pedicled NSF, harvested from the nasal septum and vascularized by sphenopalatine branches which provided a reported 5% of postoperative CSF leak rate [36].

Wormald et al. reported the “bath-plug” technique, Leng at al. defined the “gasket seal” closure, Amit et al. described the “champagne cork” technique (CCT), while recently, Cavallo et al. have reported the “3F” strategy (fat—flap—flash) [7, 27–29].

Common principles of new strategies involve (1) filling the empty dead space, (2) obtaining a watertight closure and (3) creating meticulous multilayer dural closure to reduce the entity of intra-operative CSF flow acting on the skull base defect [3, 11, 18].

In addition, different soft materials have been described for dural reconstruction, fat and ITT were the most used [3, 29]. ITT has been described as the ideal graft for dural replacement thanks to its availability, handling and strength [8]. However, also synthetic dural substitutes have been produced with the effort of miming ITT properties [3, 7]. Different ideas have been reported about rigid materials application [16, 30].

### IOL grade

According to the literature, a graded approach to skull base repair after endonasal surgery should be considered in the endoscopic era [15]. Esposito et al. classified IOL as follows: Grade 0, no leak observed; Grade 1, small leak without obvious diaphragmatic defect; Grade 2, moderate leak; or Grade 3, large diaphragmatic/dural defect [14]. Sigler at al. focused on the entity of the leak’s flow, defining the high flow leak characterized by large dural defect and basal cisterns or ventricular opening, while small dural defect and moderate CSF leak defined the low flow leak [30].

In this study, Grade 1 or 2 of the Esposito’s system were considered a low flow CSF leak (ILFL), while Grade 3 were considered a high flow leak (IHLF), as reported in other papers [31].

The literature results showed that multilayered closures could be considered essential in the reconstructive phase of EEA in both cases of ILFL and IHFL [2, 3, 12, 21]. Furthermore, NSF combined with multilayered dural reconstruction should be performed in cases of IHFL, while no differences were reported adding vascularized flap in cases of ILFL [15, 18].

In this series, IOL grade could be considered the main predicting factor for POL risk (Table 4).

POL rate, resulted higher in the IHFL group (25.5%) than in both the ILFL and INL groups (10.5% and 1.5% respectively) (Table 3). Moreover, considering single grades of IOL, the IHFL group showed higher probability to have POL (Exp(B) 16.25, p.001), while the INL group resulted in a protective factor with a higher probability to not show POL (Exp(B) 0.016, p.002).

This result could be considered as another validation of the crucial role of CSF leak flow acting on skull base osteo-dural defect and it should be carefully analyzed during reconstruction planning.

### Anatomical site and type of surgical approach

Anatomical site and type of surgical approaches still remain a critical aspect of reconstructive algorithm [32]. Indeed, higher post-operative CSF rates have been described in expanded approaches than in standard ones [33]. The extent of skull base defect should be always assessed because frontal extension or caudal extension were associated with higher risk for post-operative CSF leakage [3, 10]. Moreover, meningiomas or craniopharyngiomas were more frequent in this location and higher POL rate was described for these tumors, due to their arachnoid invasion [15]. Some authors have also described the association between POL and the size of the defect (higher risk for defect larger than 3 cm) [10, 31].

In this series, the type of IOL was found to have a key role in determining significant differences among different anatomical sites of the approach (Table 4, Fig 2). IHFL was higher in expanded approaches (88.2%) and in anterior or posterior extensions. Furthermore, POL rate increased in both standard and expanded approaches when IHFL grade was encountered (83.3% and 17.8% respectively). Even considering the anatomical site of the approach, POL was higher when IHFL grade was registered (Fig 2).

These results further highlight the importance of IOL grade in predicting POL risk, considering different surgical approaches, in order to choose the best strategy for reconstruction.

Synnderman et al. recently reviewed different reconstructive strategies for anterior and posterior cranial fossa. A three-layer reconstruction with dural substitute, fascia lata and NSF was described for the anterior cranial fossa [3, 10], while vascularized multilayer reconstruction with fat was considered mandatory for posterior fossa defects to reduce the risk of pontine herniation and also because clival tumors often needed adjuvant radiotherapy [3, 15, 18]. Sellar region was considered a low risk zone due to the low flow of CSF, if no supra-sellar extension was needed (12% of POL described). In the latter case, multilayered reconstruction and NSF were suggested [21, 34].

In this series, reconstructive strategies were prevalently chosen according to expected IOL grade.

POL rate resulted higher in type 3 reconstruction (13%) than in type 2 (6.5%) and type 1 reconstruction (1.7%). This result could be linked to the fact that type 3 reconstruction was used predominantly for procedures with larger osteo-dural defects and higher IOL grades. Moreover, in addition to the volume of the defect that could increase the POL rate, as recently described by Turri-Zanoni [10], it is well known among skull base surgeons that extended approaches and complex reconstructions were frequently adopted for more invasive and more complex malignancies (e.g. craniopharyngioma, chordoma, tuberculum-sellae meningiomas, aggressive and/or giant pituitary adenoma), which could require a more aggressive intradural dissection with consequent higher risk for high grades IOL and POL rates [15, 31]. For the abovementioned reasons, different reconstructive strategies were analyzed according to different IOL grades. Considering the IHFL group, POL rate resulted lower (4.3%) in patients undergoing a type 3 reconstruction (Table 5). This association was stronger considering fat positioning, which was the only significant factor linked to lower POL rate in IHFL group. Albeit thorough analysis of different dural substitutes was beyond the aim of the authors, the use of 2 dural layers reconstruction showed to reduce the rate CSF leak (Table 5). Moreover, although several complications related to dural substitutes positioning have been described in literature [34], namely infections and material extrusion, no complications ascribable to dural substitute issues were reported in the presented series.

**Fig 4:**
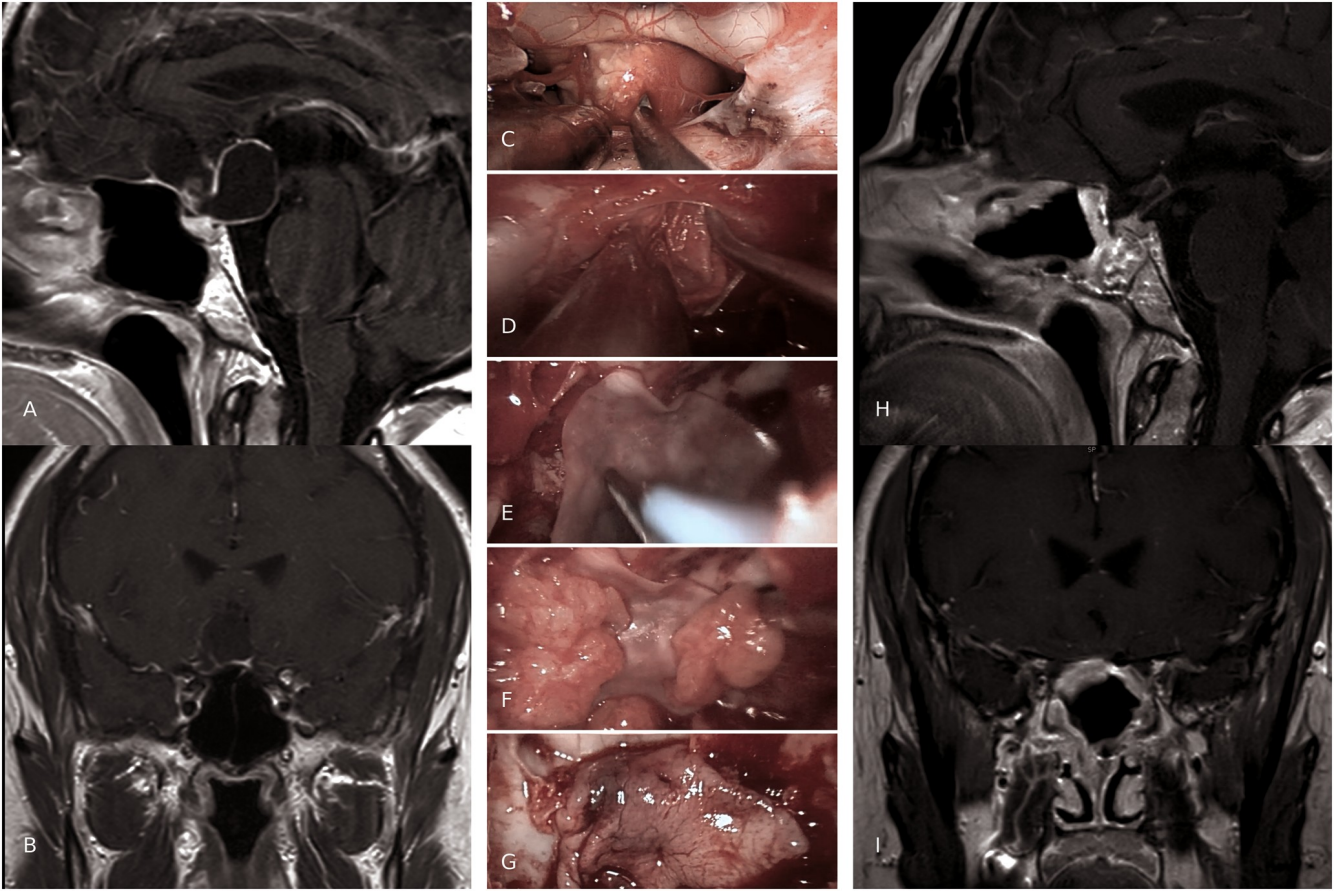
A case example of "sandwich” multilayered reconstruction. Preoperative sagittal (A) and coronal (B) MRI scans, show a T1w hypointense suprasellar 37x25 mm lesion with ring enhancement. C-G intra-operative views of reconstruction steps after a craniopharyngioma removal. C, Transtuberculum-transplanum approach for sopra-sellar pathology; note the optic chiasm pushed forward by the tumor; D, “inlay” synthetic dural substitute; E, "onlay” synthetic dural substitute, which is positioned over a first layer of autologous fat graft; F, pieces of autologous fat graft; G, NSF is fashioned over the layers previously described; three-months post-operative sagittal (H) and coronal (I) MRI scans (T1w with gadolinium), show GTR and the multiple layers adequately placed. No postoperative CSF-leaks was described in the current case.

Again, the same results were not significant in patients with both low flow or no IOL, further strengthening the role of this aspect in analyzing different strategies for multilayer reconstruction (Fig 4).

To summarize, “sandwich” multilayer reconstruction with vascularized flaps should be always considered when IHFL occurs or could be planned considering the type and the anatomical site of surgical approach. The accuracy of different layers positioning and the capability to fill the empty dead space could be considered the target, in order to reduce the CSF flow acting on the skull base defect.

### LD positioning

In this study LD was placed predominantly in patients who underwent expanded EEA, according to different IOL grade (11.8% in ILFL group, 66.6% in IHFL group).

The rate of post-operative CSF is reported in Table 5.

LD positioning during EEA has been debated in literature and no agreement has been found yet [21, 34, 35, 37–39].

Recently Zwagerman et al. published a prospective, randomized controlled trial reporting that perioperative LD, used in the contest of NSF and multilayered reconstruction, significantly reduced the rate of postoperative CSF leaks among those patients who might to have high risk of CSF leak (8,2% in LD group vs 21,2% in the control group) [37]. Nonetheless, multilayer reconstruction and firm closure of skull base defect should be mandatory in LD groups to avoid pneumocephalus sustained by unidirectional valve mechanism [37, 40].

In contrast, Eloy et al. retrospectively review 59 patients who underwent EEA repair of high flow CSF leak with NSF without LD, reporting 0% of POL rate, even if the majority of patients had sopra-sellar pathologies [38].

In this study, no significative statistic differences were found between LD and no LD group. Considering the IHFL group, POL rate resulted lower in LD positioning group (23.5% vs 29.4%), although this difference did not reach the statistical significance. Thus, no conclusions about the utility of LD positioning could be drawn from this study, even if results seem to support a contribution in reducing POL rate, especially in IHFL group.

No association between LD and meningitis was found in this study, despite the literature reports a positive correlation between longer duration of LD and higher rate of infections [41].

### Re-do surgery and radiation

Re-do surgeries and radiotherapy were considered a significant part of skull base tumors management, so their impact on POL have been analyzed in different studies [18, 42]. Nishioka et al. reported that POLs were significantly increased in case of prior trans-sphenoidal surgery or radiotherapy [42]. This could be related to bone changes and mucosal devascularization due to radiotherapy, while sphenoid scars could contribute to the higher risk described for second surgery [18]. In this series, no significative different POL rates were found between patients who underwent previous radiotherapy and/or surgery. Nevertheless, vascularized multilayer reconstruction was strongly recommended in these patients in order to provide better healing and prevent bone damage due to radiotherapy [3, 43].

### A decreasing trend through the years in preventing CSF leaks

A decreasing trend in POL rate was observed during the years (from 14.7% to 2%; p.002) (Fig 3) and these results were confirmed even stratifying patients for IOL grade and for type of surgical approach, confirming trends described from other authors [10, 44–46]. Analyzing the curve, two peaks in POL were observed, the former at the beginning and the latter in the middle of the center’s endoscopic learning curve. This feature was confirmed even in the expanded approach and in the IHFL group. The first peak could be related to the unavoidable drawbacks of acquiring a basic expertise in reaching a proper setting, an adequate affinity among the team members, other than a personal surgical experience and skills. The second peak, instead, could be explained by the following growth of the number of more complex operations (e.g. more expanded approaches). This has probably led to initial higher rates of POL, followed by a concrete reduction in the last two years. Moreover, analyzing the number of patients without POL through the years, a constant increasing trend was observed and this was confirmed also after stratification (Fig 3). These details could be related to the evolution of reconstructive strategy adopted during the years, with more tailored reconstruction in these high-risk patients, according to the expected IOL grade.

### Limitation

Principal limitation of this study is its retrospective nature. The choice of reconstructive strategy and general management were made in absence of a standardized protocol. Moreover, the adopted reconstructive strategies, summarized in Table 1, could not be considered mandatory, since they represent an indicative and no standardizable algorithm resulting from different methods adopted and lessons learned during the authors’ institution experience. Nevertheless, although the analysis does not allow to make stratified recommendations about the best reconstructive strategies, the role of risk factors and reconstructive techniques could be reliably highlighted. This was, in fact the aim of this paper.

Another limitation of this study lies in the fact that a consistent subgroup of patients who underwent minimal skull base reconstruction have been included. Nonetheless, albeit it is widely accepted among skull base surgeons that small sellar defects without intra-operative CSF leak might not need reconstruction, the risk of post-operative leak could not be considered nil among these patients. Moreover, the presence of small dural tears or coexisting independent risk factors for CSF leak could explain the reported rates of POL. Besides, although the use of fibrin glue and sphenoidal sinus mucosa are not widely considered a reconstructive strategy, the use of fibrin glue for the treatment of post-operative CSF leak have been described [47]; additionally, the rationale behind sphenoid sinus mucosa repositioning lies into the evidence of re-epithelization and subsequent coverage of the defect. Hence, from this perspective, patients treated with minimal reconstruction strategy have been included.

Nonetheless, further considerations would need a prospective analysis with the aim to analyze additional risk factors and to validate reconstructive strategies.

## Conclusion

The critical analysis of this series shows that IOL grade strongly affects POL rate. The type and the anatomical site of surgical approach should be considered during surgical planning in order to predict and assess IOL grade. No evidence of LD positioning effectiveness was found, even if it seems to be useful when IHFL was encountered. Once this aspect has been defined, tailored skull base reconstruction strategy should be chosen. Multilayer reconstruction with fat and NSF could reduce the rate of CSF leak in patients with IHFL, but the accuracy of different layers positioning and the capability to fill the empty dead space should be considered as the target. Surgical experience and skill play a role in lowering POL rate.

## Supporting information

S1 File Spreadsheet reporting all the available anonymous data of this research article. (XLSX)

## Abbreviations

CSF: Cerebro-Spinal Fluid
CVJ: Cranio-Vertebral Junction
EEA: Endoscopic Endonasal Approach
ENT: Ear-Nose-Throat
GTR: Gross Total Removal
IHFL: Intra-operative High Flow CSF Leak
ILFL: Intra-operative Low Flow CSF Leak
INL: Intra-operative No CSF Leak
IOL: Intra-operative CSF Leak
ITT: Ileo-Tibial Tract
LD: Lumbar Drain
NSF: Naso-Septal Flap
POL: Post-operative CSF Leak
